# Architectures of archaeal GINS complexes, essential DNA replication initiation factors

**DOI:** 10.1186/1741-7007-9-28

**Published:** 2011-04-28

**Authors:** Takuji Oyama, Sonoko Ishino, Seiji Fujino, Hiromi Ogino, Tsuyoshi Shirai, Kouta Mayanagi, Mihoko Saito, Naoko Nagasawa, Yoshizumi Ishino, Kosuke Morikawa

**Affiliations:** 1 Laboratory of Protein Organic Chemistry, Institute for Protein Research, Osaka University, Open Laboratories of Advanced Bioscience and Biotechnology (OLABB), 6-2-3 Furuedai, Suita, Osaka 565-0874, Japan; 2 Department of Bioscience & Biotechnology, Faculty of Agriculture and Graduate School of Bioscience & Bioenvironmental Sciences, Kyushu University, 6-10-1 Hakozaki, Higashi-ku, Fukuoka-shi, Fukuoka, 812-8581, Japan; 3 Department of Bioscience, Nagahama Institute of Bioscience and Technology, 1266 Tamura, Nagahama 526-0829, Japan; 4 Division of Structural Biology, Medical Institute of Bioregulation, Kyushu University, Maidashi 3-1-1, Higashi-ku, Fukuoka 812-8582, Japan; 5 BIRD, JST, Japan

## Abstract

**Background:**

In the early stage of eukaryotic DNA replication, the template DNA is unwound by the MCM helicase, which is activated by forming a complex with the Cdc45 and GINS proteins. The eukaryotic GINS forms a heterotetramer, comprising four types of subunits. On the other hand, the archaeal GINS appears to be either a tetramer formed by two types of subunits in a 2:2 ratio (α_2_β_2_) or a homotetramer of a single subunit (α_4_). Due to the low sequence similarity between the archaeal and eukaryotic GINS subunits, the atomic structures of the archaeal GINS complexes are attracting interest for comparisons of their subunit architectures and organization.

**Results:**

We determined the crystal structure of the α_2_β_2 _GINS tetramer from *Thermococcus kodakaraensis *(*Tko*GINS), comprising Gins51 and Gins23, and compared it with the reported human GINS structures. The backbone structure of each subunit and the tetrameric assembly are similar to those of human GINS. However, the location of the C-terminal small domain of Gins51 is remarkably different between the archaeal and human GINS structures. In addition, *Tko*GINS exhibits different subunit contacts from those in human GINS, as a consequence of the different relative locations and orientations between the domains. Based on the GINS crystal structures, we built a homology model of the putative homotetrameric GINS from *Thermoplasma acidophilum *(*Tac*GINS). Importantly, we propose that a long insertion loop allows the differential positioning of the C-terminal domains and, as a consequence, exclusively leads to the formation of an asymmetric homotetramer rather than a symmetrical one.

**Conclusions:**

The DNA metabolizing proteins from archaea are similar to those from eukaryotes, and the archaeal multi-subunit complexes are occasionally simplified versions of the eukaryotic ones. The overall similarity in the architectures between the archaeal and eukaryotic GINS complexes suggests that the GINS function, directed through interactions with other protein components, is basically conserved. On the other hand, the different subunit contacts, including the locations and contributions of the C-terminal domains to the tetramer formation, imply the possibility that the archaeal and eukaryotic GINS complexes contribute to DNA unwinding reactions by significantly different mechanisms in terms of the atomic details.

## Background

DNA replication is an essential event for all living organisms, and thus the basic mechanism is conserved from bacteria to eukaryotes. Genomic DNA replication must be executed accurately and only once during the S phase of the cell cycle. Rapid and accurate DNA replication requires the assembly of a large number of proteins, termed the replisome, which directs major reactions, such as origin recognition, template DNA unwinding, and primer extension. Accumulating evidence has identified the essential proteins for DNA replication, which have helped to provide a better understanding of the complex puzzle of DNA replication [1]. For example, in eukaryotes, a heterohexamer composed of the Mcm2-Mcm7 subunits works as the MCM helicase to unwind the template DNA, but this helicase activity is very low *in vitro *[2]. While Schwacha's group demonstrated the significant helicase activity of yeast Mcm2-7 alone [3,4], extra protein factors, which enhance the MCM helicase activity, have been found, and in particular, Cdc45, MCM, and GINS form a complex called the unwindosome or the CMG complex, which plays an essential role in the template DNA unwinding reaction, and thus this complex is considered to be the functional replicative helicase [5-8]. GINS, named from the Japanese go-ichi-ni-san, meaning 5-1-2-3, is a heterotetramer in eukaryotes, comprising the Sld5, Psf1, Psf2, and Psf3 subunits, and is essential for both initiation and elongation in DNA replication [9-11]. GINS is recruited to the replication origin with Dpb11 and Sld3 for the initiation and may functionally interact with DNA polymerase ε [9]. A functional interaction between human GINS and DNA polymerase α/primase complex is also reported [12], indicating its important role to move from initiation to elongation of the DNA replication. GINS actually forms a transient complex, designated as the preloading complex (pre-LC), with DNA polymerase ε, Sld2, and Dpb11, in a cyclin-dependent kinase (CDK)-dependent manner in budding yeast [13].

**Figure 1:**
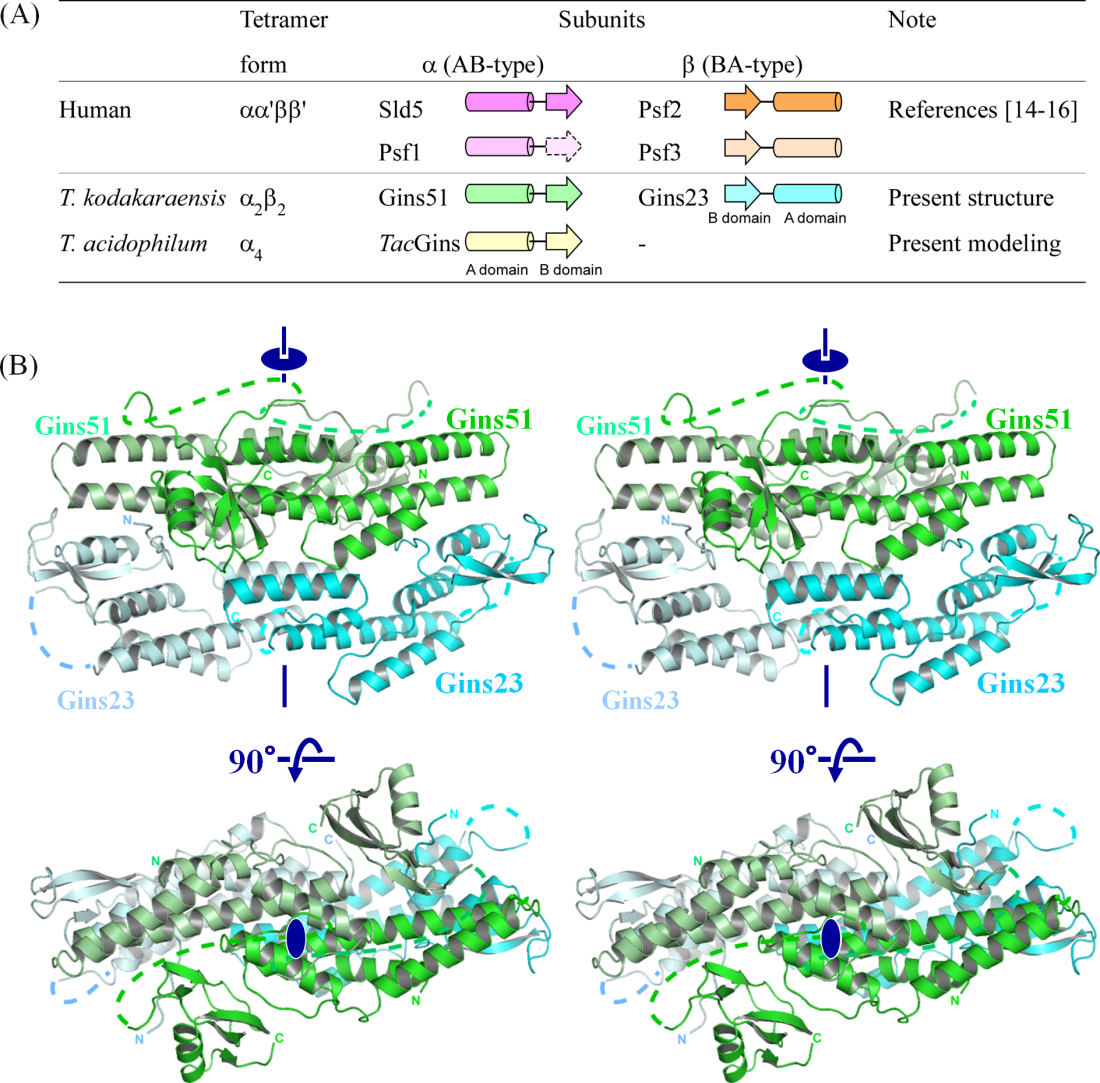
**Crystal structure of *Thermococcus kodakaraensis *GINS**. (A) Domain organization of GINS from human, *Thermococcus kodakaraensis*, and *Thermoplasma acidophilum*. Cylinders represent A-domains mainly composed of α-helices and arrows show B-domains rich in β-strands. This coloring is used throughout the manuscript. The B domain of human Psf1 is missing in the crystal structures. (B) Ribbon representation of the structure. The Gins51 subunits are colored green, and the Gins23 subunits are cyan. Missing parts are shown with dotted lines. The crystallographic two-fold axis is indicated. The figure was prepared with PYMOL http://www.pymol.org.

In 2007, three research groups reported the crystal structures of human GINS. Together, they showed a rigid and stable tetrameric core structure, with multiple flexible surface regions that are important for functional interactions with other DNA replication proteins [14-16]. Each subunit of human GINS consists of an α-helical main domain and a β-stranded small domain, and assembles into the heterotetramer, which exhibits a unique trapezoidal or ellipsoidal shape. Interestingly, the four subunits share a similar fold, in spite of the low amino acid sequence identity of at most 15%. Furthermore, they are classified into two groups. One group, including human Sld5 and Psf1, possesses the α-helical (A) domain at the N-terminus and the β-stranded one (B) at the C-terminus (this arrangement is called AB-type), and the other group, comprising human Psf2 and Psf3, is the permuted version (BA-type; Figure 1A) [17-19].

It is well known that the DNA metabolizing proteins from archaea are similar to those from eukaryotes, in both structure and function [20]. Furthermore, the archaeal DNA replication system appears to be a simplified version of the eukaryotic one. Therefore, structural studies of the archaeal system could be beneficial to understand the more complex DNA replication mechanism of eukaryotes. Bioinformatics analyses using genomic information from archaea and eukaryotes suggested that the archaeal GINS complexes are simplified versions of the eukaryotic complexes. The archaeal complexes are probably either α_2_β_2 _type tetramers composed of the two types of subunits or α_4 _type homotetramers of a single subunit, suggesting that the four different eukaryotic GINS subunits had diverged from a common ancestor [17]. The functional tetrameric assembly of the archaeal GINS has been proved by biochemical analyses. An archaeal GINS homologue was first identified in *Sulfolobus solfataricus*, as a binding partner of MCM [18]. The GINS complex from *Pyrococcus furiosus *increases the cognate MCM helicase activity, in a similar manner to eukaryotic GINS, in spite of the lack of a eukaryotic Cdc45 homolog [21]. Due to the low sequence similarity between the archaeal and eukaryotic GINS subunits, information about the three-dimensional structure of archaeal GINS complexes is crucial to obtain clearer insights into the functional subunit assembly.

In this paper, we report the crystal structure of the α_2_β_2 _GINS tetramer from *Thermococcus kodakaraensis *(*Tko*GINS), which consists of the Gins51 and Gins23 subunits. This first crystal structure of an archaeal GINS was compared with that of human GINS. The overall fold of each subunit and the tetrameric assembly of *Tko*GINS are essentially similar to those of human GINS. However, the locations of the C-terminal small domains are strikingly different between the two GINS, thus resulting in the formation of different subunit contacts in the complexes. Based on the two GINS crystal structures, we built a homology model of the putative homotetrameric GINS from *Thermoplasma acidophilum *(*Tac*GINS), and found that *Tac*GINS could form the tetrameric subunit structure, in a similar manner to the α_2_β_2 _*Tko*GINS tetramer and the human GINS heterotetramer. These results suggest that the basic function of GINS in DNA replication is conserved across the domains of life.

## Results and Discussion

### Structure of *Tko*GINS

The crystal structure of *Tko*GINS was determined by the multiple isomorphous replacement method with anomalous dispersion (MIRAS), and the atomic model was refined at 2.65 Å (Table 1). The crystal contains one pair of the Gins51 and Gins23 subunits in the asymmetric unit, and the protein forms the α_2_β_2 _tetramer generated by the crystallographic two-fold symmetry operation (Figure 1B).

**Table 1:** X-ray diffraction data collection and refinement

*Data Collection Summary*					
			Derivative		
		Native	Ta_6_Br_14_	SeMet	K_2_PtCl_4_
Wavelength	(Å)	1.0000	1.2544	0.9795	1.0717
Resolution	(Å)	50.0-2.65	50.0-3.16	50.0-2.80	50.0-3.19
(Highest shell)		(2.74-2.65)	(3.27-3.16)	(2.90-2.80)	(3.30-3.19)
Measured reflections		227882	127012	181671	112023
Unique reflections		16278 (1598)	9676 (901)	13780(1256)	9034 (921)
Completeness	(%)	99.4 (100.0)	97.6 (95.1)	99.1 (92.8)	94.5 (99.9)
*I*/σ(*I*)		17.6 (9.9)	23.8 (11.4)	14.5 (7.1)	14.2 (7.4)
Redundancy		14.0 (14.0)	13.1 (13.5)	13.2 (9.7)	12.4 (9.3)
*R*_merge_	(%)	4.3 (38.8)	5.6 (23.0)	7.5 (33.9)	6.7 (38.6)

***MIRAS Phasing Statistics***					

*R*_iso_(*F*) (%)			12.4	21.1	17.1
Number of Sites			2	6	1
Resolution	(Å)		50.0-4.0	50.0-4.0	50.0-4.0
Phasing Power (Centric/Acentric)			0.56/0.57	0.64/0.54	0.63/0.62
Figure of merit (Centric/Acen.)		0.32/0.36			

***Refinement***					
Resolution	(Å)	50.0-2.65			
*R*_work_/*R*_free_^a^	(%)	25.7/29.7			
Number of atoms					
Protein		2623			
Water		33			
Average B-factor	(Å^2^)				
Protein		65.7			
Water		56.9			
r.m.s.d.					
Bond Lengths	(Å)	0.01			
Angles	(°)	1.364			
PDB code		3ANW			

^a^*R*_merge _= (Σ|I_*I *_< I_*I *_> |)/Σ_*I *_|I_*I*_|, where < I_*I *_> is the mean I_*I *_over symmetry-equivalent reflections.

^b^*R*_work _=Σ|F_O _- F_C _|/Σ|F_O_| for all data excluding data used to calculate *R*_free_.

^c^*R*_free _was calculated using 5% of the total reflections, which were chosen randomly and omitted from the refinement.

Gins51 is composed of the larger α-helical domain at the N-terminus, and the smaller β-stranded domain at the C-terminus. In the Gins51 subunit, the long linker region between the two domains is disordered in the crystal, and 16 residues could not be assigned in the electron density map. Therefore, although we built an atomic model with the C-terminal domain contacting the nearest N-terminal one, the positions of the C-terminal domains may be swapped between the two Gins51 subunits to those in the tetrameric structure. Gins23 also possesses similar structural domains as in Gins51, but the order of the two domains in the sequence is permutated. Each domain of Gins23 can be separately superimposed on that of Gins51. The Gins23 N-terminal domain superimposed on the Gins51 C-terminal one with a root mean square deviation (RMSD) of 0.81 Å, using the corresponding 38 Cα atoms, and the Gins23 C-terminal domain superimposed on the Gins51 N-terminal one with an RMSD of 1.36 Å, using 89 Cα atoms.

### Architectural comparison between the *T. kodakaraensis *and human GINS complexes

**Figure 2:**
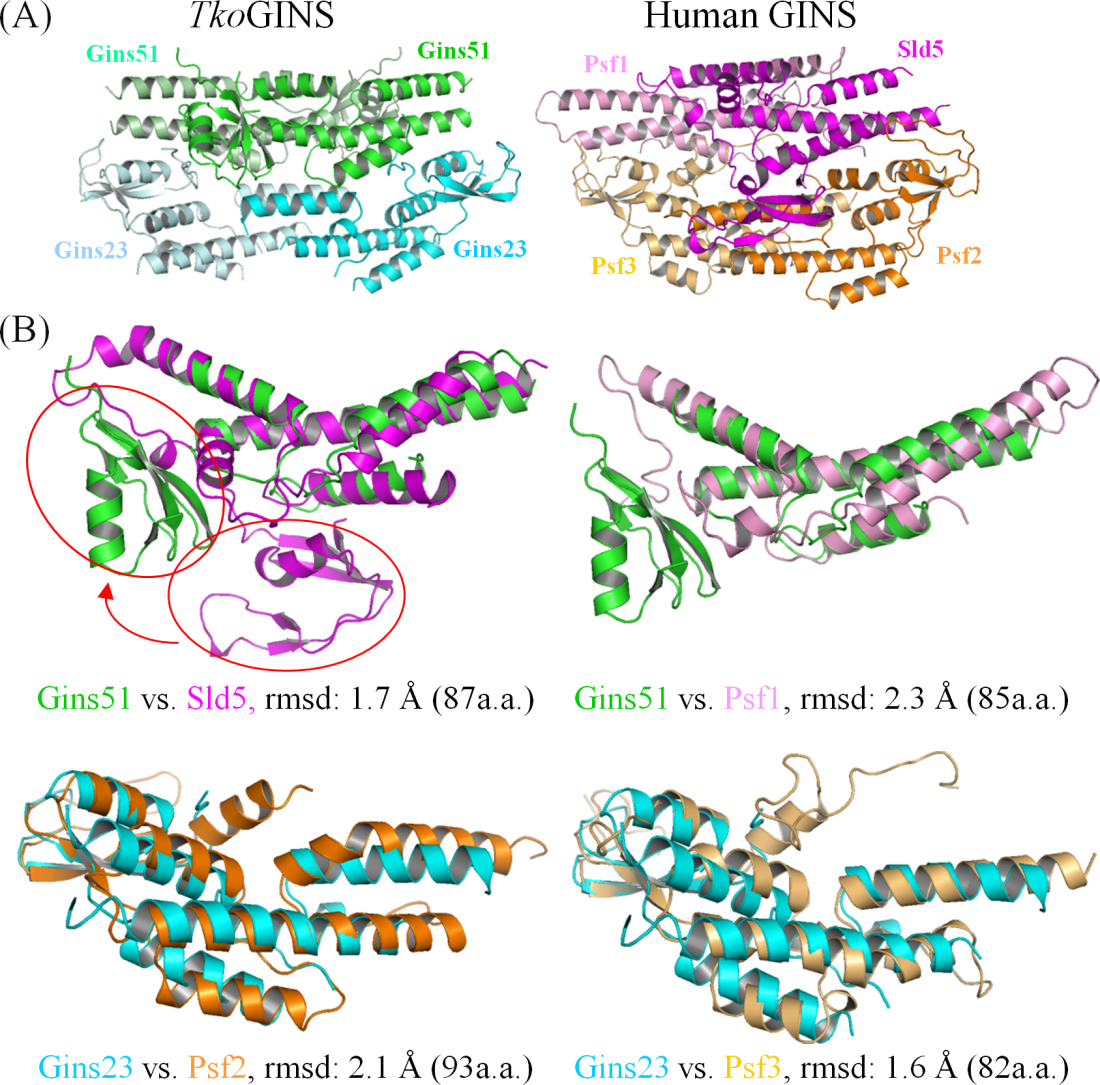
**Comparison of the archaeal and human GINS complexes**. (A) Comparison of the tetrameric structures between the *Thermococcus kodakaraensis *and human GINS complexes. (B) Comparison of the corresponding subunits.

The *Tko *GINS tetrameric structure is similar, in terms of shape and size, to the human GINS complex, comprising the four different subunits, Sld5, Psf1, Psf2 and Psf3 [14-16]. While both crystal structures contain a narrow central hole, an electron microscopic analysis of human GINS revealed a horseshoe-shaped structure with a large central hole [22]. It is tempting to speculate that GINS undergoes a conformational change between the open and closed forms, and the crystal structure exhibits a closed state. As expected, the spatial location of the *Tko*Gins51 subunit in the tetramer corresponds to those in human Sld5 and Psf1, and that of *Tko*Gins23 corresponds to those of Psf2 and Psf3 (Figure 2A). *Tko*Gins51 from the upper layer superimposed onto human Sld5 and Psf1 with RMSDs of 1.7 Å and 2.3 Å, using 87 and 85 Cα atoms in the A domains, respectively (Figure 2B, upper line). In the case of the lower layer subunits, the two Gins23 subunit structures are entirely similar to their human counterparts, including the relative locations between the two domains. Indeed, *Tko*Gins23 superimposed onto Psf2 with an RMSD of 2.1 Å using 93 Cα atoms, and onto Psf3 with an RMSD of 1.6 Å using 82 Cα atoms. These structural similarities highlight the evolutionary conservation between the archaeal and eukaryotic GINS complexes (Figure 2B, lower column). Notably, Swiatek and MacNeill reported the structural similarity between the B domains of the human GINS proteins and the C-terminal domain (CTD) of the primase small subunit (PriS)-CTD) from *Sulfolobus solfataricus *[23]. The B domains of the *Tko*GINS subunits are also similar to PriS-CTD [see Additional file 1], supporting an interesting hypothesis that archaeal PriS acquired its CTD by straightforward sequence duplication.

Despite the similarities in the internal architecture of each subunit and the overall morphology of the tetrameric subunit assembly, notable differences are observed between the archaeal and human GINS complexes. Firstly, the locations of the C-terminal B domains are strikingly different between the archaeal Gins51 and the human Sld5. In the superimposed structures, the *Tko*Gins51 B domain is located about 30 Å away from that of human Sld5 (Figure 2B). In the human complex, the B domain of Sld5 contributes to the stable tetramer formation, and the observed location in the crystal structure is in fact important for its function [15].

**Figure 3:**
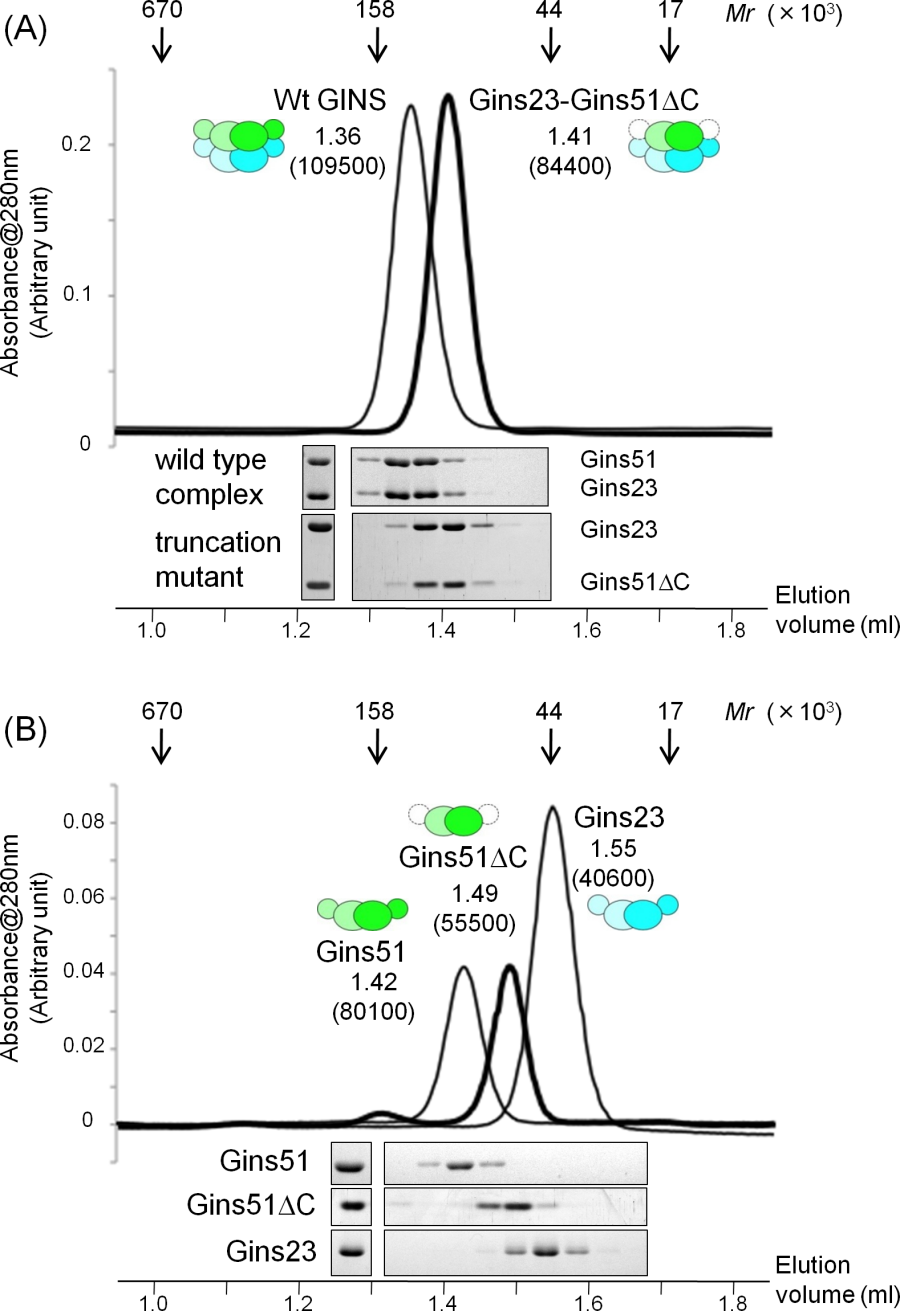
**Gel filtration chromatography of *Tko*GINS complexes**. (A) Elution profiles of the wild type (thin line) complex and a truncation mutant (thick line) complex lacking the Gins51 C-terminal domain (Gins51ΔC: Met1-Arg130) (upper part). The elution positions of the marker proteins, thyroglobulin (670 K), immunoglobulin G (158 K), ovalbumin (44 K), and myoglobin (17 K), are shown by arrows on the top. The elution volume (ml) for each molecule is indicated on the top with the apparent molecular mass shown in parenthesis. Aliquots of each fraction were subjected to 12.5% SDS-PAGE analysis followed by Coomassie Brilliant Blue staining (lower part). (B) Gel filtration chromatography and SDS-PAGE of the isolated Gins51, Gins23 and Gins51ΔC.

On the other hand, the corresponding domain of human Psf1 is highly mobile. Kamada *et al*. and Choi *et al*. crystallized the GINS proteins lacking the Psf1 B domain [14,15]. Although Chang *et al*. solved the crystal structure of the full-length GINS, they could not observe the electron density for the Psf1 B-domain in the crystal [16]. Thus, to determine whether the Gins51 B domain is required for the stable tetramer formation, we examined the oligomeric state of a *Tko*GINS truncation mutant, lacking the B domain of Gins51, by gel filtration. As shown in Figure 3A, this truncation mutant forms a stable tetramer, establishing that the C-terminal B domain of Gins51 is not essential for stable tetramer formation. Notably, the isolated Gins51 subunit forms a dimer irrespective of the presence or absence of the C-terminal B domain, and the isolated Gins23 also eluted as a dimer (Figure 3B). It is presently unclear whether the *Tko*Gins51 B domain occupies a functional position, as in the corresponding domain of human Sld5, or is incidentally fixed there, due to crystal packing [see Additional file 2]. However, the mobility of this B domain is conserved from archaea to human, and hence it seems likely that these movements of the B domain are required for the function of GINS complexes.

### Comparison of subunit contacts

**Figure 4:**
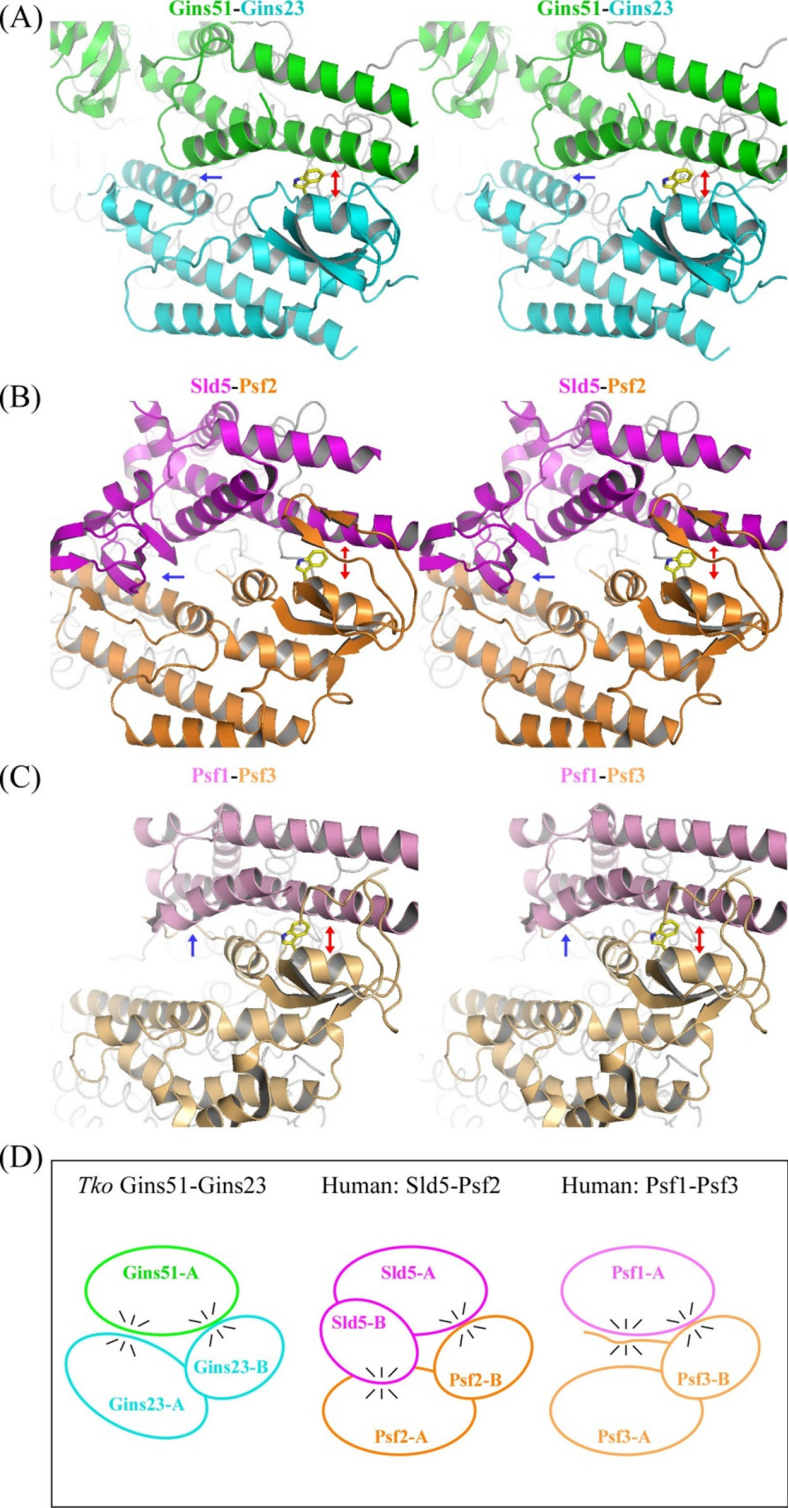
**Subunit contacts in the *Thermococcus kodakaraensis *and human GINS complexes**. Close-up views of the upper-lower layer subunit contacts, shown by stereo pairs. The conserved intermolecular helix-helix contacts observed in the first area are indicated by red arrows, and those unique in each upper-lower subunit pair in the second area are indicated by blue arrows. The conserved tryptophan residues are depicted by stick models. (A) *T. kodakaraensis *Gins51-Gins23. (B) Human Sld5-Psf2 and (C) Psf1-Psf3. (D) Schematic drawing of the subunit contacts.

A second difference was observed in the subunit contacts, particularly between the upper and lower layers (Figure 4). Overall, the archaeal Gins51-Gins51 (upper-upper) and Gins23-Gins23 (lower-lower) interactions are similar to those of the corresponding contacts, Sld5-Psf1 and Psf2-Psf3, of the human GINS, respectively. They all exhibit a similar intermolecular four α-helix bundle structure, in which two α-helices from each subunit contribute to the contact. On the other hand, the contact between Gins51 and Gins23 (upper-lower; Figure 4A) is strikingly different from the corresponding Sld5-Psf2 (Figure 4B) or Psf1-Psf3 (Figure 4C) interactions of human GINS. This difference could be attributed to the relative orientations between the upper and lower subunits.

**Figure 5:**
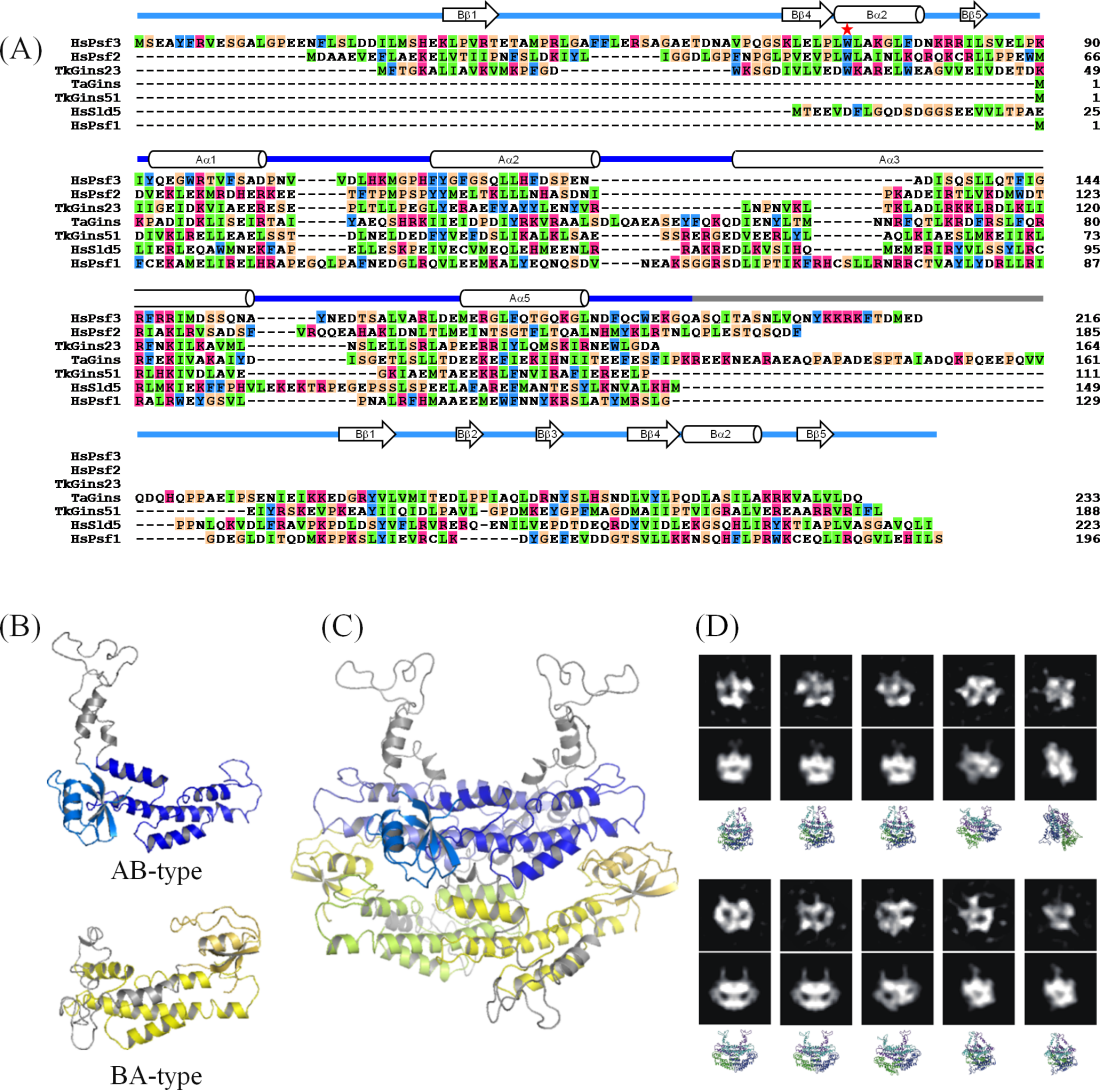
**Homology model of the *Thermoplasma acidophilum *GINS homotetramer**. (A) Structure-based sequence alignment of GINS subunits of *Homo sapiens *(Hs), *Thermococcus kodakaraensis *(Tk), and *Thermoplasma acidophilum *(Ta). Amino acid residues are colored according to their characteristics (aromatic, cyan; hydrophobic, green; basic, magenta; ambivalent, orange). Secondary structural elements are indicated on the top of the sequences. Numbering of α-helices and β-strands is according to the human GINS subunits [15]. (B) Two types of models in different conformations, the AB- and BA-types, are shown on the top and bottom, respectively. For the models, the A domain (residues 1 to 123, green in AB-type and blue in BA-type), the ID-loop (residues 124 to 172, white in both models), and the B domain (residues 173 to 233, red in AB-type and orange in BA-type) are differently colored. (C) A model of the *Tac*GINS homotetramer. (D) Class averages of electron microscopic images of the *Tac*GINS complex. The two-dimensional class averages of the EM images of the *Tac*GINS complex (upper row). Similar projections (middle row) were obtained from the assumed atomic model. The atomic models of the *Tac*GINS tetramer in the corresponding orientations are shown (lower row). The side length of the individual images in the upper and middle rows is 13.2 nm.

The contact between the upper and lower layer subunits could be divided into two areas: one involves the A domain from the upper layer subunit and the B domain from the lower layer subunit (the right side in Figure 4), while the other involves the A domain from the lower layer subunit. In *Tko*GINS, the first contact area is formed mainly between the Aα3 helix of Gins51 and the prominent Bα2 helix of Gins23, and additionally between the Aα1 helix of Gins51 and the N-terminal loop of Gins23 (Figure 4A). We found that the intermolecular helix-helix interaction is similarly observed in the human GINS structures, although the detailed contact modes, that is, the amino acid residues, contributing to the contact, are different between them [see Additional file 3]. However, a tryptophan residue is notably conserved among *Tko*Gins23 (Trp30) and human Psf2 (Trp47) and Psf3 (Trp71) in this area (Figures 4 and 5A). Our preliminary analysis suggested that this tryptophan residue is conserved among the lower layer subunits from eukaryotes and archaea (Data not shown). Therefore, it is tempting to speculate that this contact may be a key factor to generate the similar tetramer formation between the archaeal and human GINS complexes.

On the other hand, the upper-lower layer subunit contact in the second area varies among the three interactions, in terms of the relative orientations between the upper and lower subunits. *Tko*Gins23 is located close to Gins51, allowing the C-terminal Aα5 helix to interact with the Aα1 helix of Gins51 (Figure 4A). In the corresponding area of the human Sld5-Psf2 interaction, the C-terminal A domain of Psf2 is farther away from Sld5, as compared to *Tko*GINS. Instead, it is the B domain of Sld5 that faces Psf2, to form a broad contact surface (Figure 4B). This could be the reason why the Sld5 B domain in human GINS is required for stable complex formation [14]. Conversely, the corresponding B domain of *Tko*Gins51 may be dispensable for tetramer formation, because Gins51 and Gins23 can interact with each other without the Gins51 B domain. In addition, the corresponding interaction is missing between human Psf1 and Psf3, due to the high mobility of the B domain of Psf1 and the distance between the C-terminal A domains of Psf3 and Psf1. Instead, the N-terminal loop of Psf3 contacts the A domain of Psf1, but this segment is reportedly dispensable for tetramer formation [16]. Notably, *Tko*Gins23 lacks the corresponding segment at the N-terminus (Figures 4C and 5A).

The observed similarities and differences between the two-fold symmetric *Tko*GINS and the asymmetric human GINS may reflect the different evolutional processes from the last universal common ancestor. Archaea may have kept the simple subunit composition of GINS, which functions better in their simple DNA replication system. On the other hand, eukaryotes probably required the heterotetrameric GINS so that GINS can play multiple roles in their highly coordinated systems.

### Homology modeling of the *Tac*GINS homotetramer

The α_2_β_2 _*Tko*GINS and αα'ββ' human GINS complexes share a similar tetrameric assembly, in spite of the above-mentioned differences in the subunit contacts, implying that this conserved architecture is essential for the GINS functions. Thus, there is a strong interest in the structure of another type of GINS, that is, an archaeal α_4 _homotetrameric GINS. Gel filtration and electron microscopic analyses showed that *Tac*GINS indeed forms a homotetramer (Ogino *et al*., data not shown). In the absence of the three-dimensional structure of *Tac*GINS, we performed homology modeling using the present crystal structure of *Tko*GINS.

The crystal structures of the *Tko*GINS tetramers revealed a stack of a two-fold symmetric dimer of the AB-type subunits (*Tko*Gins51) onto another two-fold symmetric dimer of BA-type subunits (*Tko*Gins23). The geometric transformation linking both homodimers requires a simple translation without any rotational component. This is also the situation in the human GINS complex, although in that complex, the homodimers are heterodimers. In both cases (*Tko*GINS and the human GINS), the open interfaces at the top and bottom of the tetramer are different from those used for dimer stacking. This asymmetry is the reason why another dimer does not pile up on top of the tetramer. However, if *Tac*GINS forms a similar tetramer, composed of a single subunit, then there is no reason why it might not polymerize to form a fiber-like assembly. Indeed, in this case, the interface used for dimer-dimer stacking would also exist at the top and bottom of the tetramer and would allow other dimeric modules to assemble. The second problem is the location of the small B domain in the *Tac*GINS homotetramer. All of the GINS subunits are composed of an A domain and a B domain, but the two domains can be permutated. Sld5 and Psf1 of human GINS and Gins51 of *Tko*GINS are the AB-type, with the A domain at the N-terminus and the B domain at the C-terminus. The location of the two domains in the sequence is opposite in human Psf2 and Psf3 and *Tko*Gins23, forming the BA-type. A structure-based sequence alignment (Figure 5A) revealed that *Tac*Gins is an AB-type subunit. Thus, the question arises as to how the authentic GINS tetramer can be formed with the only AB-type subunit in the *Tac*GINS α_4 _tetramer.

The flexibility in the spatial position of the B domain relative to the A domain, which was revealed by the comparison of *Tko*GINS and human GINS, and the disorder of the interdomain loop observed in the *Tko*GINS structure could provide a reasonable solution for these problems. The solution assumes two types of structural models for the *Tac*Gins subunits (Figure 5B), which could be possible by using the long (nearly 40 amino acid residues) interdomain loop. This loop is much longer than those of the other homologous subunits and is rich in glutamic acid, glutamine, and proline residues, and therefore is likely to form an intrinsically disordered (ID) structure (ID-loop in Figure 5A). Figure 5C shows a possible homotetramer model of *Tac*GINS. This tetramer model appears to simultaneously solve the above-mentioned two problems. On the upper layer, we tentatively placed the B domains of *Tac*Gins in a similar orientation to those in the *Tko*Gins51 subunits (Figure 5B). It should be noted that other locations are possible, because as seen in the two GINS crystal structures, the B domains of the AB-type subunits could be mobile. For example, human GINS was crystallized by truncating the mobile B domain of Psf1 [14,15], and in *Tko*GINS, the Gins51 B domain is not required for stable tetramer formation (Figure 3). However, regardless of the location of the B domains, the ID-loop should protrude above the two domains, which could prevent the stacking of another dimer on the top interface (Figure 5B, AB-type). On the other hand, on the lower layer, we constructed a model in which the AB-type *Tac*Gins mimicked the domain positioning of the BA subunit (Figure 5B, BA-type). The ID-loop is long enough for this domain positioning. Taken together, the interdomain ID-loop of *Tac*Gins could play two roles in the homotetramer: to block non-functional polymerization on the tetramer surfaces, and to form a long connector to allow large domain repositioning. In other words, we propose that this long ID-loop allows the differential positioning of the B-domains and, as a consequence, exclusively leads to an asymmetric homotetramer, rather than a symmetrical one.

To experimentally validate the predicted *Tac*GINS tetramer model, we performed an electron microscopic analysis of *Tac*GINS (Figure 5D). The two-dimensional class averages thus obtained exhibited various shapes, according to the different orientations of the complex. Interestingly, a number of these class averages were very similar to the projections simulated from the predicted tetramer model.

## Conclusions

We determined the 2.65 Å resolution crystal structure of the first archaeal GINS complex from *T. kodakaraensis*, which forms a α_2_β_2 _heterotetramer composed of the Gins51 and Gins23 subunits. The overall tetrameric structure of the archaeal GINS is similar to those of the reported human GINS complexes, but the locations of the small domains and the contact modes between the subunits are different between the archaeal and human GINS complexes. The gel filtration experiment revealed that the B domain of *Tko*GINS is not required for tetramer formation. This mobility is similar to the corresponding domain of Psf1 of human GINS, but different from the other corresponding domain of Sld5 of human GINS, which is required for stable tetramer formation. These differences imply that minor variations exist in the interactions with other DNA metabolizing proteins, in terms of the atomic details. Next, we built a reasonable model of the *T. acidophilum *GINS homotetramer, which has a highly similar structure to those of the *T. kodakaraensis *and human GINS crystal structures. Taken together, these structural analyses suggest that the GINS function is essentially conserved between archaea and human. Considering this important viewpoint, an archaeal homolog of the eukaryotic Cdc45 protein, which would participate in a replicative helicase complex similar to that of eukaryotes, could exist, although it has not been identified yet, presumably because of its highly divergent sequence. The present structure and the homology model of archaeal GINS will provide a structural basis for clarification of the GINS functions, including the formation of the CMG unwindosome complex.

## Methods

### Protein expression and purification

The recombinant *Tko*GINS complex was produced by co-expression of the two genes for Gins51 and Gins23 on the different expression vectors, using the conventional *Escherichia coli *expression system. The *gins51 *and *gins23 *genes were cloned into pET21a (Amp^r^) and modified pET28 (Km^r^), respectively. These two plasmids were introduced into *E. coli *codonPlus™ (DE3)-RIL cells (Stratagene, La Jolla, CA, USA) forcibly against incompatibility, and the cells containing the two plasmids were selected on the agar plate containing both kanamycin and ampicillin. The transformation efficiency was low, but practical for further experiments. The modification of the pET28a is the replacement of the thrombin recognition sequence with the Tabaco etch virus (TEV) protease protease recognition sequence for removal of the His-tag region at the N-terminus of the target protein. The cells were grown in Luria Broth (LB) medium at 37°C to an OD_600 _of 0.5, and then the production of the *Tko*GINS complex was induced by 1 mM isopropyl-β-D-thiogalactoside (IPTG), with further cultivation at 25°C for 15 hours. The cells were harvested and disrupted by sonication in buffer A (50 mM Tris-HCl, pH 8.0, 0.1 mM ethylenediaminetetraacetic acid (EDTA), 0.5 mM dithiothreitol (DTT) , and 10% glycerol). The soluble fraction was collected by centrifugation (23 000 × *g*, 10 minutes) and then was incubated at 80°C for 20 minutes, to remove the heat-sensitive proteins derived from the host cells. The supernatant was loaded onto a hydrophobic column (HiTrap Phenyl, GE Healthcare, Hino, Tokyo, Japan) and developed with a 1.0 to 0 M linear gradient of ammonium sulfate. The pooled fraction was dialyzed against buffer B (10 mM K-phosphate, 7 mM β-mercaptoethanol, 0.05 mM CaCl_2_, and 10% glycerol), and was loaded onto a CHT-II hydroxyapatite column (Bio-Rad, Shinagawa, Tokyo, Japan)), which was developed with a linear gradient of 0.01 to 1 M K-phosphate. The fraction pool was subsequently dialyzed against buffer C (50 mM Tris-HCl, pH 8.0, 0.1 mM EDTA, 100 mM NaCl, and 10% glycerol), and was loaded onto an anion exchange column (HiTrap Q, GE Healthcare). The column was developed with a linear gradient of 0.1 to 0.6 M NaCl. The purified protein was concentrated to 20 mg/ml for crystallization. To produce the selenomethionine (SeMet) derivative of *Tko*GINS, the *E. coli *cells harboring the expression plasmids were grown in minimal medium containing seleno-L-methionine, at a final concentration of 25 μg/ml. The SeMet protein was expressed and purified using the same procedure as for the wild type complex.

The preparation of the recombinant *Tac*GINS protein will be described elsewhere (Ogino *et al*.). Briefly, the gene encoding the protein (Ta1042) was cloned into the pET21a vector, and was overexpressed in *E. coli *BL21 codonPlus™ (DE3)-RIL cells. The harvested cells were disrupted by sonication, and then the soluble fraction was heated at 60°C for 20 minutes. The heat-stable fraction was treated with polyethyleneimine to remove the nucleic acids, and then was subjected to hydrophobic (HiTrap Phenyl) and anion exchange (HiTrap Q) column chromatographies.

### Crystallization, data collection, and model refinement of *Tko*GINS

Initial crystallization screening of *Tko*GINS was performed by the hanging-drop vapor diffusion method at 293 K, using a Mosquito nanodrop dispenser (TTP Labtech, Cambridge, MA, USA), followed by manual optimization. In the initial screening, 200 nl aliquots of the protein solution were mixed with an equal volume of reservoir solutions from commercially available screening kits, JCSG Core 1 to 4 (Qiagen, Chuo, Tokyo, Japan). *Tko*GINS crystallized under nine conditions out of the 384 in the semi-automatic setup. An optimization procedure yielded the best diffraction quality crystals, which were obtained by mixing 1 μl of the protein solution with an equal volume of a reservoir, containing 0.1 M 4-(2-hydroxyethyl)-1-piperazineethanesulfonic acid (HEPES) (pH 7.5), 0.2 M magnesium chloride and 24% to approximately 26% (v/v) PEG400. The crystals belonged to the space group *P*4_1_2_1_2, with unit cell dimensions *a *= *b *= 74.3 Å, *c *= 181.3 Å, and contained one Gins51 subunit and one Gins23 subunit per asymmetric unit. The Se-Met protein was crystallized by essentially the same procedure as for the wild-type *Tko*GINS. Tantalum (Ta_6_Br_14_)- and platinum (K_2_PtCl_4_)- derivatized crystals were produced by adding a tiny amount of the heavy-atom water solution into the crystallization drop. Due to the presence of a high concentration of PEG400 and glycerol in the crystallization drop, no additional cryo-protectant was required. Crystals were picked up from the crystallization drop and directly flash-cooled in the nitrogen gas stream at 100 K for X-ray diffraction data collection. All data sets were collected on BL38-B1 of SPring-8 (Harima, Japan) and processed by the HKL2000 package [24].

Structure determination by molecular replacement, using the human GINS structure, was unsuccessful, even though many subunit combinations were tested as probes. Therefore, the structure was determined experimentally by the MIRAS method, using the above three derivatives. All of the heavy atom sites were located on isomorphous or anomalous Patterson maps, and the heavy atom parameters were refined by the program SHARP [25]. The phases were improved by density modification techniques, with the programs DM and SOLOMON in the CCP4 suite [26]. The atomic model was built with the program O [27], and the crystallographic refinement was performed with the program CNS [28]. Manual remodeling and refinement were iterated until satisfactory convergence was achieved. The final atomic model contained residues 1 to113, 129 to 186 of Gins51, seven amino acids derived from the N-terminal tag, residues 1 to 93, 101 to 131, 134 to 164 of Gins23, and 33 water molecules. Residues 114 to 128 and 187 to 188 of Gins51 and residues 94 to 100 and 132 to 133 of Gins23 were missing in the electron density map. All non-glycine and proline residues are located in either the most favored, additionally allowed, or generously allowed regions of the Ramachandran plot (data not shown). The crystallographic analyses are summarized in Table 1. The atomic coordinates of *Tko*GINS have been deposited in the Protein Data Bank, under the accession code 3ANW.

### Gel filtration chromatography of the purified *Tko*GINS

Gel filtration chromatography was performed to analyze the oligomeric states of the *Tko*GINS subunits and the complexes, using a SMART system and a Superdex 200 PC 3.2/30 column (GE Healthcare). The column was equilibrated with buffer D (50 mM Tris-HCl, pH 8.0, 0.15 M NaCl, 7 mM β-mercaptoethanol, 0.1 mM EDTA). A 20 μl aliquot of each protein (20 μM) was applied to the column, which was developed with buffer D. Aliquots of the fractions were subjected to 12.5% SDS-PAGE analysis followed by Coomassie Brilliant Blue staining. A Bio-Rad gel filtration standard was used to estimate the molecular weights of the GINS complexes.

### Homology modeling of *Tac*GINS

The structure-based alignment was made by using the program MATRAS [29]. The homology model of *Tac*GINS was constructed by using the Homology module of the MOE application (Ryouka Systems, Inc., Chuo, Tokyo, Japan), which was based on the methods of Levitt [30] and Fechteler *et al*. [31]. First, the amino acid sequence of *Tac*Gins (conserved hypothetical protein of *T. acidophilum*; accession code: CAC12170) was aligned with *Tko*Gins51, as shown in Figure 5A, and a homology model was generated for the AB-type subunits, according to the sequence alignment (Figure 5B). In order to model the BA-type subunits, the sequences of the N- and C-terminal (A and B) domains of *Tac*Gins were separately aligned with the C- and N-terminal domains of *Tko*Gins23, respectively, and the domains were connected with the ID-loop sequence of *Tac*Gins.

When the subunits of *Tac*GINS were assembled into a tetramer, by superposing them onto each corresponding subunit of the *Tko*GINS tetramer, the ID-loop of the BA subunits exhibited extensive atomic clashes with the upper layer subunits. Therefore, the ID-loop was manually rebuilt, to avoid atomic clashes. Finally, the *Tac*GINS tetramer was energy minimized, in order to remove other atomic clashes among the subunits (Figure 5C).

### Electron microscopy and single particle image analysis of *Tac*GINS

The purified *Tac*GINS complex was suspended in a buffer containing 50 mM Tris-HCl (pH 8.0) and 40 mM NaCl, (final protein concentration: 5 μg/ml). A 3 μl aliquot of the sample solution was applied to a copper grid supporting a continuous thin-carbon film, left for 1 minute, and then stained with three drops of 2% uranyl acetate. Images of molecules were recorded by an Eagle 2 K CCD camera (FEI, Hillsboro, OR, USA) with a pixel size of 2.76 Å/pixel, using a Tecnai T20 electron microscope (FEI) operated at an accelerating voltage of 200 kV. A low dose system was used to reduce the electron radiation damage of the sample. A total of 1,989 images of the *Tac*GINS complex were selected, using the BOXER program in EMAN [32]. The two-dimensional class averages were obtained using the refine2d tool of EMAN, assuming 50 classes. The simulated two-dimensional projection maps were calculated from the atomic model of the *Tac*GINS tetramer, obtained by homology modeling.

## Abbreviations

MCM: minimichrosome mentenance; Cdc: cell division cycle; Sld: synthetic lethal with dpb11; Psf: partner of Sld 5; Dpb: DNA polymerase B; pre-LC: preloading complex; CDK: cyclin-dependent kinase; MIRAS: multiple isomorphous replacement method with anomalous dispersion; RMSD: root mean square deviation; CTD: C-terminal domain; PriS: primase small subunit; ID: intrinsically disordered; Amp: ampicillin; Km: kanamycin; TEV: Tabaco etch virus; LB: Luria broth; IPTG: isopropyl-β-D-thiogalactoside; EDTA: ethylenediaminetetraacetic acid; DTT: dithiothreitol; HEPES: 4-(2-hydroxyethyl)-1-piperazineethanesulfonic acid; PEG: polyethylene glycol.

## Authors' contributions

TO performed the crystallization and structure determination, and wrote the manuscript. SI, SF, and HO performed the protein expression, purification, and biochemical analyses. NN assisted with the crystallization. MS performed the homology modeling. KMa performed the electron microscopy and helped to write the manuscript. TS designed the homology modeling and helped to write the manuscript. YI and KMo conceived of the study and wrote the manuscript. All authors read and approved of the final manuscript.

## Supplementary Material

Additional file 1 **Structure comparison of the *Tko*GINS B domains with the C-terminal domain of the primase small subunit (PriS-CTD) from *Sulfolobus solfataricus ***(PDB code 1ZT2** chain A)**. (A) Stereo view of the superimposed structures. *Tko*Gins51 B domain is colored green, *Tko*Gins23 B domain is cyan, and Pris-CTD is pink. The Gins23 B domain superimposed on the Gins51 B domain with an RMSD of 0.81 Å, using the corresponding 38 Cα atoms, and the PriS-CTD superimposed on the Gins51 B domain with an RMSD of 0.85 Å, using 31 Cα atoms. (B) Structure-based sequence alignment. Click here for file

Additional file 2 **Crystal packing interactions**. (A) Overall view of the crystal packing. Each tetramer contacts the surrounding four tetramers in the crystal with the same interaction mode. (B) Close-up view of the packing interaction boxed in (A). A Gins51 B domain contacts a Gins23 B domain in the neighboring tetramer. Click here for file

Additional file 3 **Detailed subunit contacts in the GINS complexes**. Close-up views of the subunit contacts between Gins51 and Gins23 in *Tko*GINS (A), Sld5 and Psf2 in human GINS (B), and Psf1 and Psf3 in human GINS(C) are shown by stereo pairs. Residues involved in the contacts are depicted with stick models. (D) to (F) Schematic representations of the contacts. Click here for file
