# Supplementary figures and images for: Architectures of archaeal GINS complexes, essential DNA replication initiation factors
Source: BMC Biol. 2011 Apr 28;9:28. doi: 10.1186/1741-7007-9-28 (PMC3114041; doi:10.1186/1741-7007-9-28)

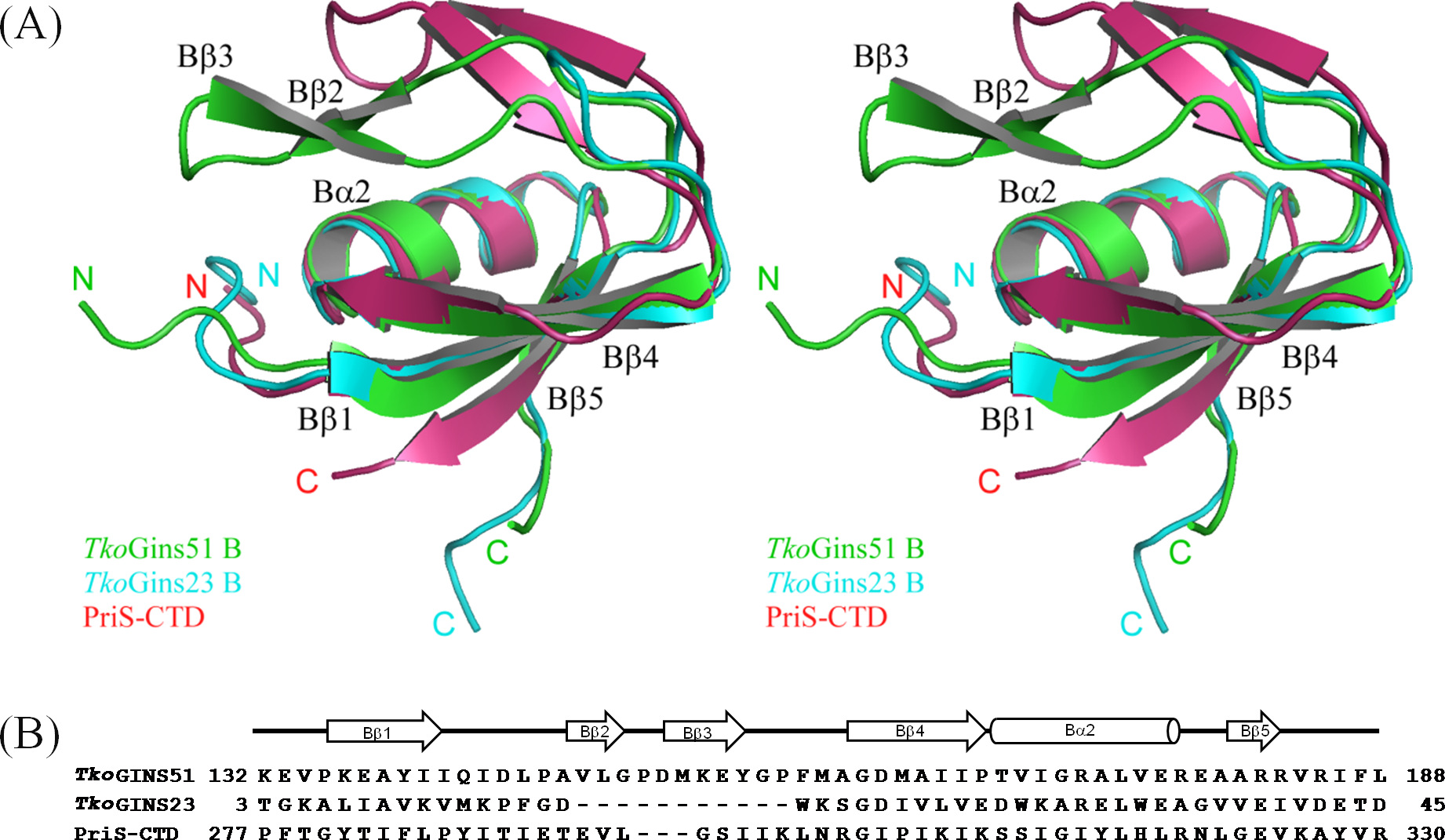

Supplement: Additional file 1 — Structure comparison of the TkoGINS B domains with the C-terminal domain of the primase small subunit (PriS-CTD) from Sulfolobus solfataricus (PDB code 1ZT2 chain A). (A) Stereo view of the superimposed structures. TkoGins51 B domain is colored green, TkoGins23 B domain is cyan, and Pris-CTD is pink. The Gins23 B domain superimposed on the Gins51 B domain with an RMSD of 0.81 Å, using the corresponding 38 Cα atoms, and the PriS-CTD superimposed on the Gins51 B domain with an RMSD of 0.85 Å, using 31 Cα atoms. (B) Structure-based sequence alignment. [file 1741-7007-9-28-S1.PNG]

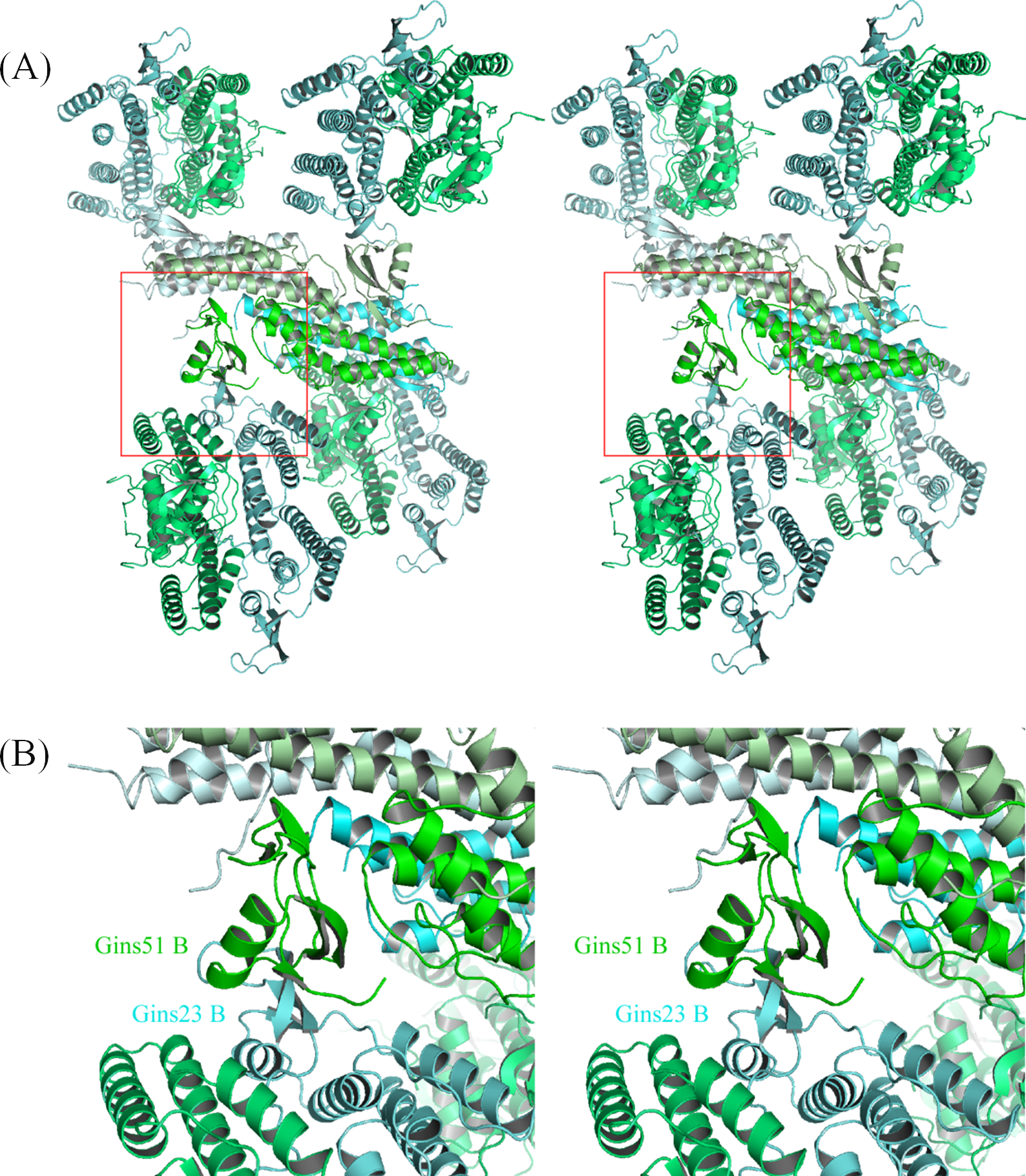

Supplement: Additional file 2 — Crystal packing interactions. (A) Overall view of the crystal packing. Each tetramer contacts the surrounding four tetramers in the crystal with the same interaction mode. (B) Close-up view of the packing interaction boxed in (A). A Gins51 B domain contacts a Gins23 B domain in the neighboring tetramer. [file 1741-7007-9-28-S2.PNG]

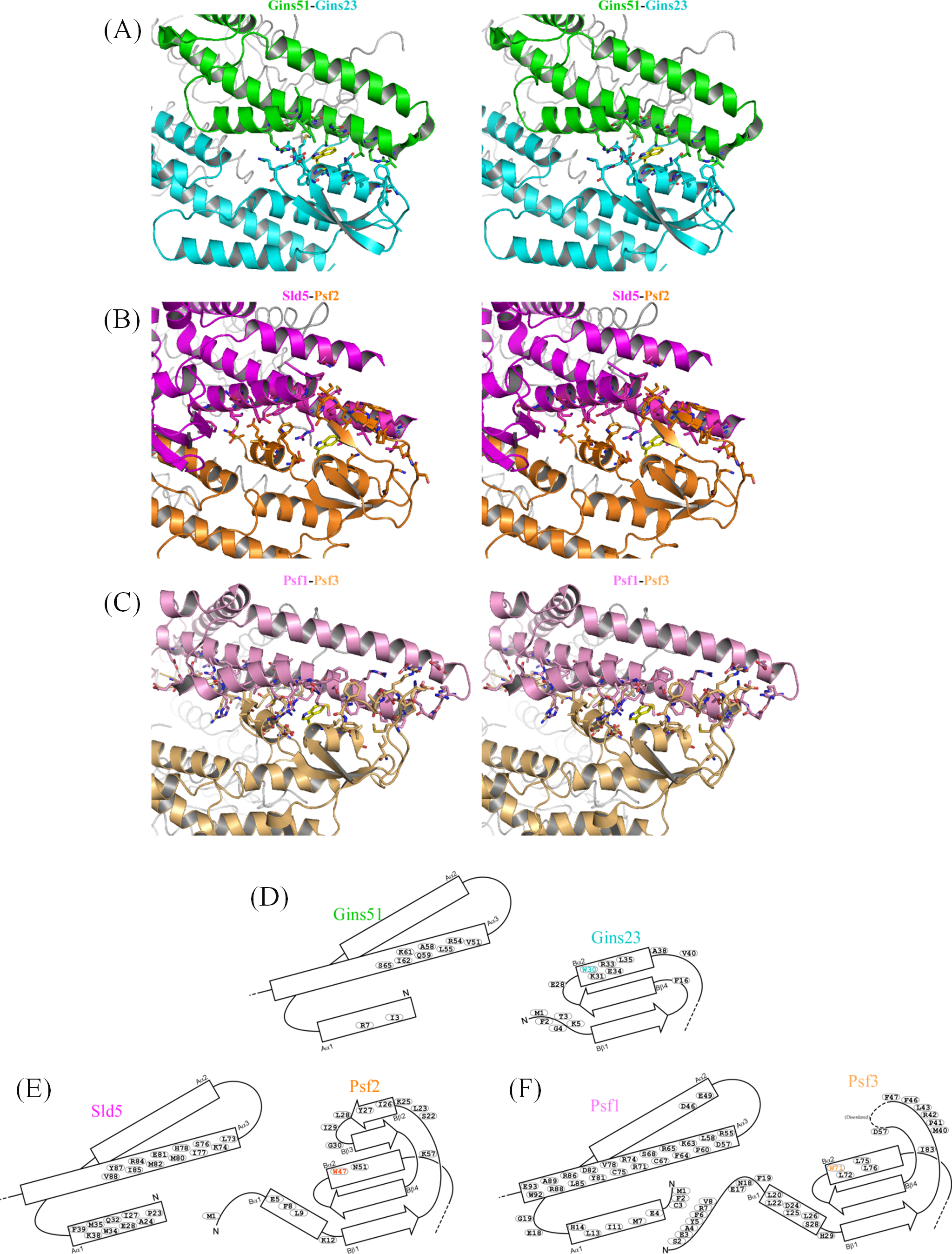

Supplement: Additional file 3 — Detailed subunit contacts in the GINS complexes. Close-up views of the subunit contacts between Gins51 and Gins23 in TkoGINS (A), Sld5 and Psf2 in human GINS (B), and Psf1 and Psf3 in human GINS(C) are shown by stereo pairs. Residues involved in the contacts are depicted with stick models. (D) to (F) Schematic representations of the contacts. [file 1741-7007-9-28-S3.PNG]
